# Reducing inappropriate and unnecessary implantable cardioverter-defibrillator therapy: Is patient confirmation via a mobile application the solution?

**DOI:** 10.1016/j.hroo.2023.11.004

**Published:** 2023-11-10

**Authors:** Alwin B.P. Noordman, Michiel Rienstra, Yuri Blaauw, Robert Tieleman, Alexander H. Maass

**Affiliations:** ∗ Department of Cardiology, University Medical Center Groningen, University of Groningen, Groningen, the Netherlands; † Department of Cardiology, Martini Hospital, Groningen, the Netherlands

**Keywords:** Ventricular tachycardia, Mobile application, Smartwatch, Implantable cardioverter-defibrillator, Self-management

**Key findings**

▪ Implantable cardioverter-defibrillator therapies can be unnecessary, for instance when a patients is hemodynamically stable in the presence of an otherwise spontaneously terminating ventricular tachycardia.
▪ A proposed novel mobile application that alerts patients that a shock or antitachycardia pacing will be delivered within a predefined time interval unless a button is clicked may provide the solution to reducing inappropriate and unnecessary therapies.
▪ The mobile application could be aided by a plethysmograph measuring patients’ blood pressure to help determine their hemodynamic state.
▪ Prior to the implementation of the proposed hypothetical mobile application in clinical practice, extreme care should be taken to guarantee patient safety by involving all relevant stakeholders in the development of the mobile application.

## Background

Implantable cardioverter-defibrillators (ICDs) have been shown to be associated with a reduction of all-cause mortality mostly driven by reduction of sudden cardiac death (SCD).^1^^,^^2^ However, a substantial number of patients receive inappropriate therapies, delivered in the absence of potentially life-threatening ventricular arrhythmias. Modern ICD programming has led to a reduction of inappropriate shocks to 7% in 4 years.^3^ Besides being painful and a potential source of anxiety, inappropriate shocks are potentially arrhythmogenic and can even increase the risk of death.^4^ In this creative concept, we propose a novel idea that could potentially be implemented in the future to reduce the number of inappropriate and unnecessary ICD therapies.

## Unnecessary therapies

ICD therapies are generally defined as appropriate or inappropriate. Some patients might benefit from early delivery of ICD therapies, since too long a delay might result in patients losing consciousness and potentially lead to physical injury as well as a loss in bodily confidence. In other patients, ICDs may deliver therapies too soon, for instance in the presence of ventricular tachycardias (VTs) that would otherwise have terminated spontaneously and when a patient is still hemodynamically stable and experiences little or no symptoms. Current strategies imply that rate is the major determinant of hemodynamic instability, whereas many more factors are of importance, such as underlying cardiac function, ventricular activation patterns, and hemodynamic demand at the moment of occurrence of the arrhythmia. It is thought that less than one-third of ICD therapies is life-saving.^5^ The Multicenter Automatic Defibrillator Implantation Trial-Reduce Inappropriate Therapy (MADIT-RIT) study demonstrated that delayed therapy led to fewer first and total occurrences of appropriate therapy.^6^ This could indicate that VTs may terminate spontaneously without the requirement of ICD therapy, as has been shown to be the case for approximately 35% of VT episodes.^7^ Similarly, the Primary Prevention Parameters Evaluation (PREPARE) study, which applied detection prolongation through a higher number of intervals to detect, found fewer shocks, without an increase in untreated VTs and arrhythmic syncope.^8^ These results support the notion that, in the case of spontaneously terminating VTs, the delivery of a shock or antitachycardia pacing (ATP) would be unnecessary. Furthermore, ATP can in itself be proarrhythmic, converting hemodynamically stable VT into faster VT or ventricular fibrillation. Therefore, a third category of ICD therapies exists in addition to appropriate and inappropriate therapies, namely that of unnecessary therapies.

## Potential unexplored solutions

There is a need to reduce the number of inappropriate and unnecessary therapies. Part of the solution is ICD programming. To prevent inappropriate therapy for atrial fibrillation with fast ventricular conduction, higher rates are programmed and discriminators are set in place. In order to differentiate between supraventricular tachycardia (SVT) and VT, morphology of near-field and far-field intracardiac electrograms can be analyzed by the ICD. In addition to programming high rates and rhythm discriminators, modern ICD programming is often set to a number of intervals to detect of 30.^9^ Although higher than in the past, this means that most therapies occur within 10 seconds. The ENHANCED Implantable Cardioverter Defibrillator programming to reduce therapies and improve quality of life (ENHANCED-ICD) study demonstrated that a further delay was safe and could prevent unnecessary therapies in a considerable number of patients.^10^

In addition to ICD programming, remote monitoring plays an important role, as it can help to reduce the number of inappropriate shocks.^11^ In fact, the current guidelines recommend remote monitoring for this reason.^12^

A check for hemodynamic stability of the detected arrhythmia would be the ultimate discriminator between appropriate and inappropriate or unnecessary ICD therapies. To achieve this, several techniques are envisioned. The patient could be involved in checking hemodynamic effects of the tachycardia or the ICD or an additional tool could be used to (semi)quantitatively analyze changes in blood pressure. Closed-loop stimulation is used as a sensor for rate response but could also be tested for hemodynamic consequences of tachycardias.^13^ The analysis of finger plethysmography or impedance cardiography would involve an additional device that communicates with the ICD, but these techniques have been incorporated into daily clinical practice, and testing their use would be limited to communication with the ICD and definition of thresholds to withhold therapy. In our view, patient involvement constitutes the most promising and easiest additional discriminator, which may be useful for withholding unnecessary and inappropriate ICD therapies. In the end, if still conscious, the patient can be instructed to assess their situation for symptoms of hemodynamic instability, such as dizziness.

## Mobile applications in cardiac care

A thus far unexplored strategy concerns patient confirmation through a mobile application. Mobile phones and smartwatches have become commonplace. This brings opportunities for health advancement. Mobile applications are already used in cardiac care. They have been found to be useful for several different patient populations, for instance for home-based cardiac rehabilitation via a smartphone in post–myocardial infarction patients,^14^ and for telemonitoring in patients with heart failure,^15^ with smartwatches and a mobile application being used in the detection of atrial fibrillation.^16^^,^^17^ Mobile applications can also be used for remote monitoring of pacemakers and ICDs, which showed a higher transmission success rate as compared with traditional forms of remote monitoring.^18^

## Proposed novel mobile application as a solution

**Figure 1:**
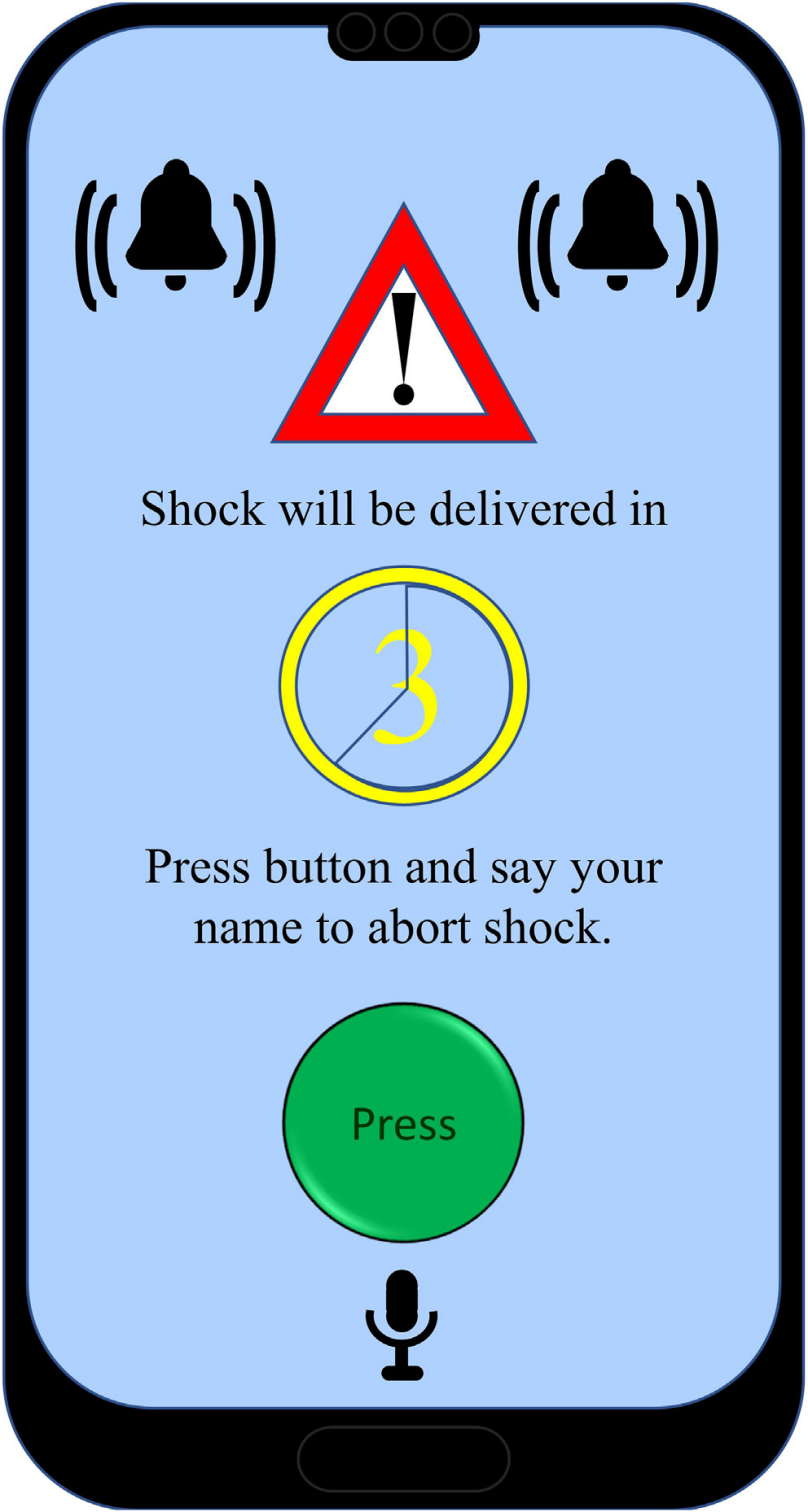
A hypothetical screen design of the proposed patient confirmation mobile application.

**Figure 2:**
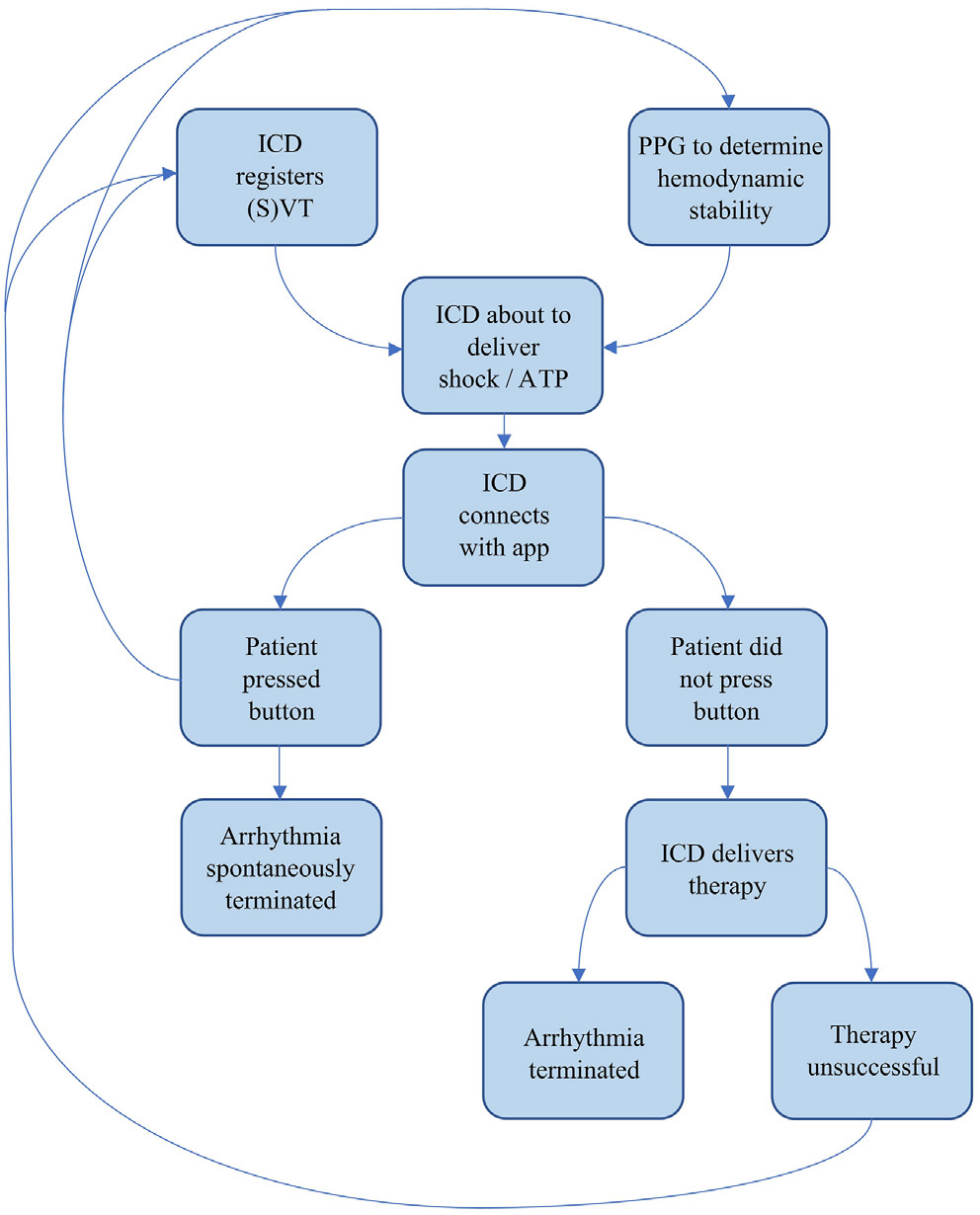
Schematic displaying the potential role of the mobile application, photoplethysmography, and patient involvement in the case of an arrhythmia. ATP = antitachycardia pacing; ICD = implantable cardioverter-defibrillator; PPG = photoplethysmography; (S)VT = (supra)ventricular tachycardia.

**Figure 3:**
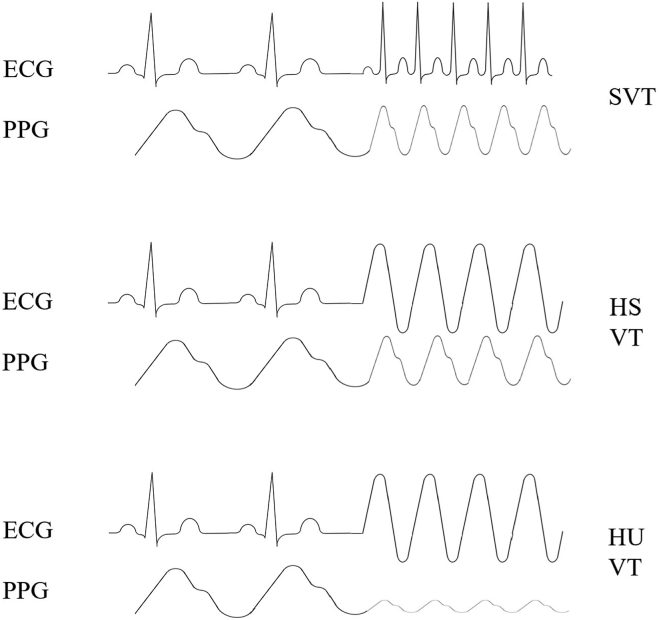
Several types of arrhythmias as detected by the implantable cardioverter-defibrillator and the arterial blood pressure as measured by photoplethysmography. ECG = electrocardiography; HS = hemodynamically stable; HU = hemodynamically unstable; PPG = photoplethysmography; SVT = supraventricular tachycardia; VT = ventricular tachycardia.

A mobile application may also be of great benefit to patients with an ICD. The role that we envision the application to have is as follows: when the ICD is about to deliver a shock or ATP, whether appropriate or inappropriate, it will first signal this to the application on the smartphone or, alternatively, smartwatch of the patient. Through an alarm, the mobile application will alert the patient that ATP or shock will be delivered within a predefined time interval unless they click a button (Figure 1). If the patient is hemodynamically compromised, they will not click the button, resulting in the ICD delivering its programmed therapies. If the patient is hemodynamically stable, they will click the button, preventing the occurrence of inappropriate or unnecessary therapy (Figure 2). In order to further prevent unnecessary therapies, blood pressure could be measured (semi)quantitatively by a smartwatch functioning as a plethysmograph, the data of which could be used by the ICD to determine whether or not the patient is hemodynamically stable (Figure 3). The plethysmograph could alternatively be implanted at the time of ICD implantation or even incorporated into the ICD itself as an additional feature, which would result in continuous blood pressure registration. However, plethysmography in this context would not be helpful in patients with a left ventricular assist device, although this concerns only a small number of patients. The time during which therapy is withheld could be programmable, with the alert recurring for instance every 10 seconds during the first minute, every minute during the ensuing 9 minutes, and subsequently every 5 minutes during the ensuing hour to check whether the patient is still conscious. The mentioned time intervals may need to vary according to the VT cycle length: shorter for faster VTs and longer for slower VTs. In addition, patients with a preserved ejection fraction could tolerate longer and faster VTs than those with (severely) compromised systolic ventricular function. In the moments after the patient clicked the button to withhold ICD therapy, plethysmography could also be helpful and be used to monitor the hemodynamic state of the patient. In the case of hemodynamic stability, no further alerts will be required. The recurring alerts would then only be necessary if the patient is hemodynamically unstable as determined by plethysmography, in which case the patient will not withhold therapy, unless there is a defect in the plethysmography system. Plethysmography may therefore obviate the need for patients to frequently click the button. A timeout could be programmed to deliver therapy even if the patient is actively trying to withhold therapy if the arrhythmia is faster than a certain programmed heart rate. If the patient transitions to a cardiac arrest, the ICD will still deliver a shock or ATP. Additionally, after a certain programmable period, the smartphone could send an automated call to the emergency services without the need for the patient to make the call. The location of the patient could be transmitted by GPS coordinates.

## Advantages of the mobile application

The proposed mobile application, which currently is still hypothetical, has several potential advantages. First, it may reduce the incidence of inappropriate and unnecessary shocks and hence their deleterious effects, especially considering the fact that delaying therapy through ICD programming resulted in the prevention of unnecessary therapies in 10% of patients in a previous study.^10^ Second, it could promote self-management, with ICD recipients having more control over their own treatment, although this may be highly patient specific. Third, delaying ICD therapy will allow extra time for hemodynamically stable VTs to terminate, likely reducing the number of unnecessary shocks. Fourth, by allowing ICDs to be programmed to also target slower VTs, which may sometimes be hemodynamically poorly tolerated, this application may prevent inappropriately withheld ICD therapy. Fifth, but not the least important, patients get the chance to maneuver themselves into a safe situation, for instance if the ICD is about to deliver therapy while driving or when the patient is in an unsafe position such as when standing on a ladder.

## Disadvantages of the mobile application

In addition to the advantages, there are also several important potential disadvantages that should be discussed. One of the disadvantages is that patients must click the button within a certain time frame, which means that they should have their smartphone with them at all times. A smartwatch would be easier to use in this regard. In certain situations, such as while driving, an alarm may distract the patient, leading to potentially dangerous situations. Careful consideration should be given to potential solutions to this problem. In addition, it is important that patients are mentally healthy and stable, because some patients may be apprehensive of withholding ICD therapy and may experience anxiety as a result. Furthermore, patients potentially have to click the button multiple times to avert inappropriate shocks. Hypothetically, a bystander could potentially click the button when therapy is in fact needed. Fingerprint recognition would not solve this problem, because a malevolent bystander could take the finger of the patient, potentially by force, and use it to click the button. Voice authentication, whereby a patient speaks to the mobile application to verify their identity, would be a potential solution. It is of the utmost importance that appropriate measures be taken to prevent inappropriate use of the application. Indeed, security issues may be one of the largest obstacles for implementation of this mobile application in clinical practice, with lack of security guidelines and lack of expertise for the development of secure mobile applications posing important challenges.^19^ It is also essential to involve all relevant stakeholders in the development process, including patients.^19^^,^^20^

## Similar concepts already existent in clinical practice

Software transferring some control over the occurrence of appropriate therapies to patients already exists. Whereas the herein proposed application provides patients with the opportunity of averting inappropriate and unnecessary therapies, currently existing software allows patients to self-administer appropriate ATP for intra-atrial re-entrant tachycardia with 1:1 conduction. This software has been shown to successfully convert such episodes.^21^ In a similar way, a mobile application may be of great benefit to patients with ventricular arrhythmias. In fact, patient interaction already exists in this context: the wearable cardioverter-defibrillator allows the patient to abort a shock. For this, the patient must press 2 buttons simultaneously.^22^ In this way, patient involvement has been shown to prevent inappropriate therapies.^23^ Compliance has been shown to be satisfactory, with more than half of patients wearing the wearable cardioverter-defibrillator at least 90% of the time.^24^ An application for remote monitoring, through which patients can also send transmissions themselves if instructed to do so, currently exists for certain ICD and cardiac resynchronization therapy defibrillator devices as well as implantable loop recorders. For the latter group, the use is already episode based, as patients activate it with symptoms but without transmission from the device to the app. The application collects data from the implanted device and sends it to the clinic. This application could potentially be used also for the newly proposed purpose, so as to offer patients more control in their own therapy. Furthermore, there are many mobile health applications that generally show positive effects. For instance, depending on the type of application, they can increase patient knowledge and quality of life.^25^

## Necessary steps prior to implementation

Several steps need to be taken before our proposed hypothetical application can be used in clinical practice. Manufacturers have to be motivated to develop the application. All stakeholders, including cardiologists, ICD technicians, device vendors, regulatory and privacy authorities, experts in the field of cyber security, and patients, must be involved in the development of the mobile application. Special care must be taken to reduce the risk of the mobile application being hacked by criminals. Especially the involvement of cyber security specialists is essential to ensure that the mobile application is safe to use for patients. As is the case for ICDs, which themselves are also vulnerable to cyberattacks, cybersecurity continues to evolve as technology continues to advance.^26^^,^^27^ A detailed analysis of the impact of the application on patients, their quality of life, and safety and security issues should be conducted. Eventually, prospective studies are needed to prove reduction of inappropriate and appropriate therapies. Such studies could include eligible relatively healthy patients who have an indication for the implantation of an ICD and randomize them to 2 groups. In both groups, patients would receive an ICD, but in only one of the groups will the mobile application be implemented as an intervention. Such a clinical trial could find out whether patients in the mobile application group experience fewer appropriate and inappropriate ICD therapies, while also investigating patient safety and user-friendliness. In addition, the optimal time interval between alarm onset and delivery of therapy as well as the optimal number of and time interval between the following repeated alerts should be determined. This is highly patient specific. Cardiac function and VT cycle length are important determinants of hemodynamic compromise. It is also important to clarify the experience of patients with the application, especially with regard to its disadvantages. Once the developmental process involving manufacturers and all other relevant stakeholders and proof-of-concept studies have been completed, further improvements should be applied to the mobile application based on the findings from studies investigating patient safety, security, effectiveness, user-friendliness, patient experience, and patient suitability criteria. Another important step to consider prior to the clinical implementation of the mobile application concerns any necessary certification and approval by the relevant regulatory institutions. Once all these steps have been completed, indications for the use of the proposed mobile application should be described in the relevant guidelines, after which follows implementation in clinical practice.

## Future perspectives

Perhaps, in the not so distant future, smartphones and smartwatches will be intricately connected with the ICD so that they become an inseparable part of it, at least for those patients who are most likely to benefit from the mobile application. At least initially, physicians should carefully select patients who are relatively healthy and fit, both physically and mentally, because not all patients may be suitable for the implementation of the mobile application in their care, given some of the aforementioned potential disadvantages. Ultimately, however, most, if not all, patients should be able to use the application, especially considering the fact that the result of not clicking the button would not be any different from the current situation, as the ICD would then deliver its therapies. There is a role for the cardiologist to determine the optimal programming strategy on a patient-specific level. However, this does not necessarily mean additional visits to the cardiologist, considering that changes in programming may only be required in the case of an inappropriate or unnecessary shock, at which time a hospital visit is currently already common.

## Conclusion

Although ICDs constitute an important part in the fight against SCD, the problem of inappropriate and unnecessary ICD therapies remains. A mobile application that alerts patients that a shock or ATP will be delivered within a predefined time interval unless a button is clicked may be able to reduce the number of ICD therapies as well as their associated morbidity and mortality.
